# An Old Friend

**Published:** 1921-01-29

**Authors:** 


### AN OLD FRIEND.

A NEW edition of Dr. J. K. Watson's popular "Handbook for Nurses" will be published early in February by The Scientific Press, Ltd., 28 and 29 Southampton Street, Strand, London, W.C. 2. The work has been entirely rewritten, and will contain over 250 additional pages and a large number of new illustrations. The price will be 10s. 6d. net, a very modest sum in view of the high prices prevailing at present for printed books of any sort.

